# A simple and rapid approach for screening of SARS-coronavirus genotypes: an evaluation study

**DOI:** 10.1186/1471-2334-5-87

**Published:** 2005-10-18

**Authors:** Grace TY Chung, Rossa WK Chiu, Jo LK Cheung, Yongjie Jin, Stephen SC Chim, Paul KS Chan, YM Dennis Lo

**Affiliations:** 1 Centre for Emerging Infectious Diseases, The Chinese University of Hong Kong, Prince of Wales Hospital, Hong Kong; 2 Department of Chemical Pathology, The Chinese University of Hong Kong, Prince of Wales Hospital, Hong Kong; 3 Department of Microbiology, The Chinese University of Hong Kong, Prince of Wales Hospital, Hong Kong

## Abstract

**Background:**

The Severe Acute Respiratory Syndrome (SARS) was a newly emerged infectious disease which caused a global epidemic in 2002–2003. Sequence analysis of SARS-coronavirus isolates revealed that specific genotypes predominated at different periods of the epidemic. This information can be used as a footprint for tracing the epidemiology of infections and monitor viral evolution. However, direct sequencing analysis of a large number of clinical samples is cumbersome and time consuming. We present here a simple and rapid assay for the screening of SARS-coronavirus genotypes based on the use of fluorogenic oligonucleotide probes for allelic discrimination.

**Methods:**

Thirty SARS patients were recruited. Allelic discrimination assays were developed based on the use of fluorogenic oligonucleotide probes (TaqMan). Genotyping of the SARS-coronavirus isolates obtained from these patients were carried out by the allelic discrimination assays and confirmed by direct sequencing.

**Results:**

Genotyping based on the allelic discrimination assays were fully concordant with direct sequencing. All of the 30 SARS-coronavirus genotypes studied were characteristic of genotypes previously documented to be associated with the latter part of the epidemic. Seven of the isolates contained a previously reported major deletion but in patients not epidemiologically related to the previously studied cohort.

**Conclusion:**

We have developed a simple and accurate method for the characterization and screening of SARS-coronavirus genotypes. It is a promising tool for the study of epidemiological relationships between documented cases during an outbreak.

## Background

The Severe Acute Respiratory Syndrome (SARS) is a recently emerged infectious disease which led to a global epidemic between 2002 and 2003. A novel coronavirus (SARS-CoV) was identified as the causative agent [1]. The genomic sequence of the SARS-CoV was promptly characterized [2,3]. Thereafter, studies had focused on the early detection of SARS-CoV and the development of diagnostic tools [4-6]. Systematic analysis of the SARS-CoV sequence information have demonstrated that characteristic viral genotypes predominated at certain periods during the course of the outbreak [7-10]. Furthermore, characterization of the viral sequences have been shown to be a useful tool for confirming epidemiological associations between infected individuals as suspected from conventional epidemiological investigations [9-11]. In-depth analysis of the available sequence data on SARS-CoV also revealed that the viral isolates could be readily subclassified into several major genotypes based on nucleotide variations at specific genomic positions [8,12]. In a large-scale phylogenetic analysis of SARS-CoV sequences [8], a 5-nucleotide motif at the GZ02 [GenBank :AY390556] reference nucleotide residues 17,564, 21,721, 22,222, 23,823, and 27,827 was identified to be most useful for distinguishing the major SARS-CoV genotypes. These major viral genotypes predominated at different periods of the epidemic [8]. Thus, it is evident that viral sequence and molecular epidemiological data provide valuable information and tools for our combat against infectious diseases. However, direct sequencing of viral isolates from a large number of clinical samples is cumbersome and time consuming. Therefore, a rapid system for the characterization and screening of viral genotypes, such as for SARS-CoV, would potentially be useful. In this study, we demonstrate the feasibility of the adoption of allelic discrimination assays based on the use of fluorogenic oligonucleotide probes for the genotyping of SARS-CoV isolates.

## Methods

### Study population

Viral culture isolates from 30 SARS patients who were admitted to the hospitals of the New Territories East Cluster of Hong Kong during the SARS epidemic were retrieved. The study was approved by the Institutional Review Board. SARS was confirmed in all cases either by positive reverse transcription-polymerase chain reaction (RT-PCR) detection of SARS-CoV RNA in clinical specimens or documented seroconversion.

### Genotype analysis by Taqman allelic discrimination assay

We focused on the development of allelic discrimination assays for the five previously described characteristic single nucleotide variations (SNV) [8]. RNA was extracted from viral isolates cultured from SARS patients' clinical specimens using the QIAamp viral RNA mini kit (Qiagen, Valencia, CA, USA), according to manufacturer's instructions. Eleven microliters of the extracted viral RNA was reverse transcribed by Superscript III (Invitrogen, Carlsbad, CA, USA) with random hexamer according to manufacturer's instructions. Genotyping of the five SNVs was determined using TaqMan (Applied Biosystems, Foster City, CA, USA) allelic discrimination assays on an ABI Prism 7900HT sequence detection system (Applied Biosystems). Each assay consisted of two allele-specific minor groove binding probes associated with either, 6-carboxyfluorescein (FAM) or VIC™ as the fluorescent label, for the discrimination of the two respective alleles at each SNV locus. One assay was designed for each of the 5 SNVs. The primer and probe sequences, designed using the Primer Express 2.0 software (Applied Biosystems) are listed in Table 1. The probes were designed such that the discriminatory nucleotide is placed close to the middle portion of the oligonucleotide. The assays were set up according to the manufacturer's instructions (TaqMan Core PCR Kit; Applied Biosystems) in a reaction volume of 25 μL. Each reaction consists of 1X Buffer A, 4 mM MgCl_2_, 0.2 μM dATP, 0.2 μM dCTP, 0.2 μM dGTP and 0.4 μM dUTP, 900 nM forward and reverse primers, 200 nM of each fluorescent probe, 0.25U UNG, 0.625U *Taq *polymerase and 0.5 μl of cDNA as template. The thermal profile consists of an initial incubation at 50°C for 2 min, and then a denaturation period at 95°C for 10 min, followed by 40 cycles of denaturation at 92°C for 15 s, and 1 min of combined annealing and extension at 60°C. The genotypes were scored with the SDS2.1 software.

**Table 1:** Primers and probes

	Sequence (all sequence starts from the 5' end)
SNV 17564*	
Forward primer	GACACTGTGAGTGCTTTAGTTTATGACA
Reverse primer	CCTTTGTAGAACATTTTGAAGCATTG
Probes	FAM-AGCTGACTTATCCTTGTGT
	VIC-AGCTGACTTCTCCTTGTGT
Synthetic template for allele T	GTTGACACTGTGAGTGCTTTAGTTTATGACAATAAGCTAAAAGCACACAAGGATAAGTCAGCTCAATGCTTCAAAATGTTCTACAAAGGTGT
Synthetic template for allele G	GTTGACACTGTGAGTGCTTTAGTTTATGACAATAAGCTAAAAGCACACAAGGAGAAGTCAGCTCAATGCTTCAAAATGTTCTACAAAGGTGT
	
SNV 21721	
Forward primer	CCATTTTATTCTAATGTTACAGGGTTTCA
Reverse primer	TTTCTCTGTGGCAGCAAAATAAATAC
Probes	FAM-ATACGTTTGGCAACCCTGTC
	VIC-ATACGTTTGACAACCCTGTC
Synthetic template for allele G	CTTCCATTTTATTCTAATGTTACAGGGTTTCATACTATTAATCATACGTTTGGCAACCCTGTCATACCTTTTAAGGATGGTATTTATTTTGCTGCCACAGAGAAATCA
Synthetic template for allele A	CTTCCATTTTATTCTAATGTTACAGGGTTTCATACTATTAATCATACGTTTGACAACCCTGTCATACCTTTTAAGGATGGTATTTATTTTGCTGCCACAGAGAAATCA
	
SNV 22222	
Forward primer	GAGCCATTCTTACAGCCTTTTTA
Reverse primer	GCCAACAAAATAGGCTGCAG
Probes	FAM-TGCTCAAGACACTTGGG-MGB
	VIC-TGCTCAAGACATTTGGG-MGB
Synthetic template for allele C	GCCATTCTTACAGCCTTTTTACCTGCTCAAGACACTTGGGGCACGTCAGCTGCAGCCTATTTTGTTGGCTATTTAAAGCCAACTACATTTATGCTCAAGTATGATG
Synthetic template for allele T	GCCATTCTTACAGCCTTTTTACCTGCTCAAGACATTTGGGGCACGTCAGCTGCAGCCTATTTTGTTGGCTATTTAAAGCCAACTACATTTATGCTCAAGTATGATG
	
SNV 23823	
Forward primer	TCGCTCAAGTCAAACAAATGTACA
Reverse primer	GAGGGTCAGGTAATATTTGTGAAAAATT
Probes	FAM-CCAACTTTGAAATATTTTGG
	VIC-CAACTTTGAAAGATTTTGG
Synthetic template for allele T	TGTTCGCTCAAGTCAAACAAATGTACAAAACCCCAACTTTGAAATATTTTGGTGGTTTTAATTTTTCACAAATATTACCTGACCCTCTAA
Synthetic template for allele G	TGTTCGCTCAAGTCAAACAAATGTACAAAACCCCAACTTTGAAAGATTTTGGTGGTTTTAATTTTTCACAAATATTACCTGACCCTCTAA
	
SNV 27827	
Forward primer	TCATTGTTTTGACTTGTATTTCTCTATGC
Reverse primer	CTTCAAGCACATGAGGTTTATTAGATG
Probes	FAM-TTGCATATGCACTGTAGT
	VIC-TTGCATACGCACTGTAGT
Synthetic template for allele C	TTCTCATTGTTTTGACTTGTATTTCTCTATGCAGTTGCATATGCACTGTAGTACAGCGCTGTGCATCTAATAAACCTCATGTGCTTGAAGATCC
Synthetic template for allele T	TTCTCATTGTTTTGACTTGTATTTCTCTATGCAGTTGCATACGCACTGTAGTACAGCGCTGTGCATCTAATAAACCTCATGTGCTTGAAGATCC

* probes for SNV 17564 are anti-sense sequences

### Sequence confirmation by direct sequencing

All viral sequences were confirmed by direct sequencing. RT-PCR was performed to specifically amplify genomic segments of SARS-CoV encompassing each of the 5 SNVs using primers and protocols previously described [7]. The DNA of each amplicon was sequenced by the dideoxy terminator method on an automated DNA sequencer (3100 Genetic Analyzer, Applied Biosystems) based on capillary electrophoresis.

## Results and discussion

**Figure 1:**
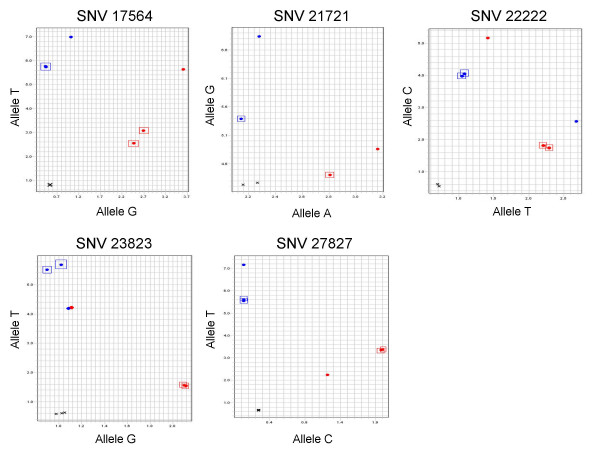
**Allelic discrimination plot of CUHK-Su10 and CUHK-W1. **Allelic discrimination at each of the 5 studied SNVs described in the text as demonstrated using the synthetic templates and cDNA from CUHK-Su10 and CUHK-W1 Vero cell culture isolates is presented in the successive plots. () synthetic template for the FAM-labeled allele, () synthetic template for the VIC-labeled allele, () CUHK-W1, () CUHK-Su10, () no template control.

Taqman allelic discrimination assays for the 5 SNVs were first tested on synthetic templates (Sigma Genosys, Australia) (Table 1) and verified using 2 viral isolates, CUHK-W1 [GenBank :AY278554] and CUHK-Su10 [GenBank :AY282752]. CUHK-W1 is a SARS-CoV isolate with a G:A:C:T:C motif at the GZ02 reference nucleotide residues 17,564, 21,721, 22,222, 23,823, and 27,827, characteristic of SARS-CoV strains isolated before worldwide dissemination of SARS [8,13]. On the other hand, CUHK-Su10 demonstrates a T:G:T:T:T motif which is characteristic of SARS-CoV strains isolated after global spread was evident. As evident from figure 1, the newly developed allelic discrimination assays were able to differentiate the 2 viral isolates and genotype each SNV correctly (Table 1).

**Figure 2:**
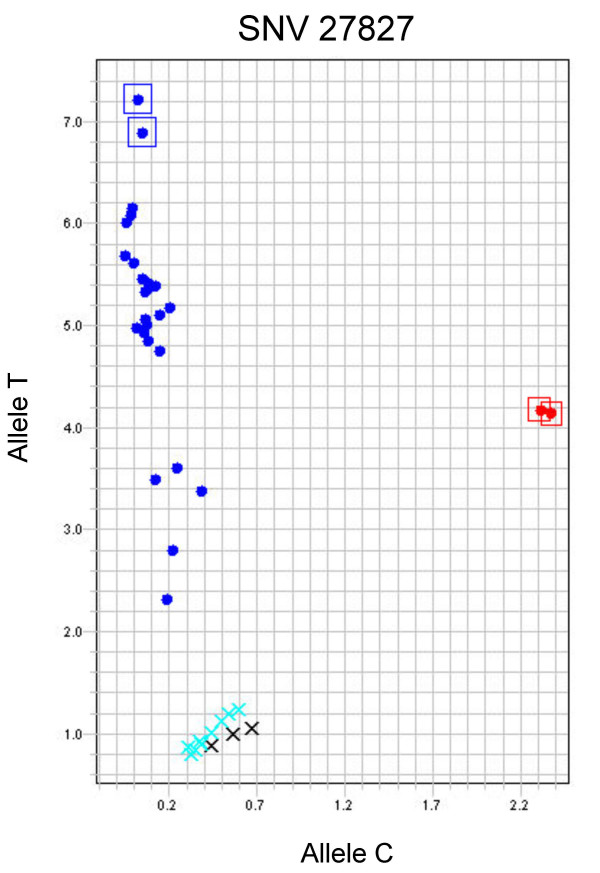
**Allelic discrimination plot of 30 SARS patients using the allelic discrimination assay on SNV 27827. **() synthetic template for FAM-labeled allele, () synthetic template for VIC-labeled allele, () patients with SARS-CoV showing a T nucleotide at SARS-CoV SNV 27827, () patients with SARS-CoV showing no allelic signal at SNV 27827. This SARS-CoV variant was confirmed to contain a 386 nt deletion encompassing SNV 27827, () no template control.

Following initial development and optimization, the allelic discrimination assays were used to genotype SARS-CoV in clinical samples. We were able to successfully determine the SARS-CoV genotypes in all 30 samples. Genotypes of virus isolates at the 5 SNV positions are shown in Table 2. SARS-CoV from all but seven cases showed the T:G:T:T:T motif resembling that of the CUHK-Su 10 isolate. For the remaining seven cases, no allelic signal was detected at SNV position 27,827 (Figure 2), leading to a T:G:T:T:/ motif.

**Table 2:** Genotype of SARS-CoV culture isolates from 30 patients determined by Taqman Allelic Discrimination assays

Case number	Nucleotide positions				
	
	17,564	21,721	22,222	23,823	27,827
TC01	T	G	T	T	T
TC02	T	G	T	T	T
TC07	T	G	T	T	T
TC10	T	G	T	T	T
TC11	T	G	T	T	T
TC14	T	G	T	T	T
TC19	T	G	T	T	T
TC20	T	G	T	T	T
TC22	T	G	T	T	T
TC26	T	G	T	T	T
TC27	T	G	T	T	T
TC28	T	G	T	T	T
TC29	T	G	T	T	ND
TC30	T	G	T	T	T
TC32	T	G	T	T	ND
TC34	T	G	T	T	T
TC37	T	G	T	T	T
TC38	T	G	T	T	T
TC39	T	G	T	T	T
TC41	T	G	T	T	T
TC42	T	G	T	T	T
TC43	T	G	T	T	T
TC44	T	G	T	T	T
TC45	T	G	T	T	ND
TC46	T	G	T	T	ND
TC48	T	G	T	T	T
TC51	T	G	T	T	ND
TC52	T	G	T	T	T
TC55	T	G	T	T	ND
TC59	T	G	T	T	ND
					
CUHK-W1 [AY278554]	G	A	C	T	C
CUHK-Su10 [AY282752]	T	G	T	T	T

ND: no allelic signal

The SARS-CoV genotypes isolated from the 30 patients were also confirmed by direct sequencing. The sequencing results were fully concordant with that based on the allelic discrimination assays at all the 5 SNVs. The seven samples which gave no allelic signal by the allelic discrimination assay at SNV 27,827 showed a shortened amplicon encompassing the region. Direct sequencing of this short amplicon revealed a deletion of 386 nt identical to a SARS-CoV deletion variant previously reported by our group [8,9]. This deletion variant was first isolated from a discrete cohort of 15 epidemiologically related SARS patients [9]. In the previous cohort of patients, the origin of the deletion variant was traceable to mid-April 2003 in two patients residing in an estate, T, in Hong Kong with subsequent spread predominantly at the North District Hospital, Hong Kong [9]. To further determine if the newly identified cases were epidemiologically related to the original patient cohort, the case histories were reviewed.

The seven patients had fever onset between April 4 to 15, 2003 which predated the disease onset dates of all cases in the original patient cohort. All but one patient presented to hospital A initially for other medical conditions without fever or evidence of chest infection and appeared to have acquired SARS nosocomially during admission. The remaining patient was a resident at Estate T and presented to hospital A with symptoms and signs of chest infection on April 8, 2003 and SARS was subsequently confirmed. Thus, it appeared that we have identified another cohort of patients harboring the SARS-CoV variant with the 386-nt deletion. It is interesting to note that this study provided additional anecdotal evidence pointing to Estate T as a propagation site for the deletion variant. In addition, we were able to trace the emergence of this deletion variant to early April 2003, weeks before the first appearance reported previously [9].

Our study has clearly demonstrated the feasibility of using allelic discrimination assays as a method for genetic characterization of SARS-CoV genotypes in patients. It is particularly useful when there is already extensive sequence information. Direct sequencing is still the gold standard for identifying new sequence variations when new agents of infectious disease continue to emerge and old ones reemerge. Once the variations have been identified, allelic discrimination assay is more efficient and suitable for large-scale population investigations. A recent study illustrated the use of mass spectrometry-based technology in characterizing SARS sequence variations [14]. However, this method requires post-PCR manipulations and the availability of specialized equipment. On the other hand, allelic discrimination assays have been widely used in the study of associations between single nucleotide polymorphisms and diseases such as cancers [15] and rheumatoid arthritis [16]. The validity of the approach for single nucleotide polymorphism genotyping has been previously demonstrated [17-19]. Thus, this study further extended the usefulness of allelic discrimination approach based on fluorogenic oligonucleotide probes. The approach provides a rapid and simple means to accurate genotype screening, making it ideal for epidemiological investigations.

## Conclusion

We have evaluated a rapid approach for characterizing SARS-CoV genotypes. The assay is simple, easy to perform and reproducible. It can therefore be used as an efficient means to screen for virus genotypes and track the transmission of a particular viral strain in times of epidemics. Incidentally, we identified a previously reported deletion variant of the SARS-CoV in a new cohort of patients and traced the emergence of this variant to an earlier date than previously reported.

## List of abbreviations

SARS: Severe acute respiratory syndrome

SARS-CoV: SARS-coronavirus

RT-PCR: reverse transcription-polymerase chain reaction

SNV: single nucleotide variation

## Competing interests

YMDL, RWKC and SSCC have filed patent applications on aspects concerning the genomics and detection of the SARS-coronavirus.

## Authors' contributions

GTYC, RWKC and YMDL have contributed in the preparation of the manuscript and the overall study design. GTYC, RWKC and YJ have contributed in the assay designs, data analysis and conducting the genotyping experiments. RWKC, SSCC, JLKC and PKSC have contributed in the collection and analysis of clinical data from the patients. JLKC and PKSC provided the viral samples.

## Pre-publication history

The pre-publication history for this paper can be accessed here:


